# Discrete time survival analysis machine learning models for IVF prognosis across multiple cycles

**DOI:** 10.1093/hropen/hoag085

**Published:** 2026-09-23

**Authors:** TingTing Shao, Achilleas Ghinis, Eline Dancet, Johanna Devroe, Felipe Kenji Nakano, Celine Vens

**Affiliations:** Department of Public Health and Primary Care, KU Leuven Campus Kulak Kortrijk, Kortrijk, Belgium; Department of Public Health and Primary Care, itec, imec Research Group at KU Leuven, Kortrijk, Belgium; Department of Public Health and Primary Care, KU Leuven Campus Kulak Kortrijk, Kortrijk, Belgium; Department of Public Health and Primary Care, itec, imec Research Group at KU Leuven, Kortrijk, Belgium; Department of Public Health and Primary Care, KU Leuven, Leuven, Belgium; Department of Obstetrics and Gynecology, Leuven University Fertility Centre, University Hospital Leuven, Leuven, Belgium; Laboratory of Endometrium, Endometriosis & Reproductive Medicine, Department of Development and Regeneration, KU Leuven, Leuven, Belgium; Department of Public Health and Primary Care, KU Leuven Campus Kulak Kortrijk, Kortrijk, Belgium; Department of Public Health and Primary Care, itec, imec Research Group at KU Leuven, Kortrijk, Belgium; Computer Science Department, State University of Londrina, Londrina, Brazil; Department of Public Health and Primary Care, KU Leuven Campus Kulak Kortrijk, Kortrijk, Belgium; Department of Public Health and Primary Care, itec, imec Research Group at KU Leuven, Kortrijk, Belgium

**Keywords:** IVF/ICSI outcome, machine learning, survival analysis, discrete time, embryo utilization rate, endometrial thickness

## Abstract

**STUDY QUESTION:**

Can discrete time survival analysis machine learning (ML) methods provide reliable and accurate predictions of cumulative live birth rate (CLBR) for patients both at diagnosis (pre-treatment) and after the first cycle of treatment (post-treatment)?

**SUMMARY ANSWER:**

We developed discrete time survival analysis ML models that provide personalized CLBR estimates of up to six complete IVF cycles with good discriminative performance, especially at the post-treatment phase.

**WHAT IS KNOWN ALREADY:**

The first relevant prognostic models to estimate IVF cumulative success rate across multiple cycles have moderate performance, with C-statistics ranging from 0.7 to 0.73 for both pre-treatment and post-treatment models.

**STUDY DESIGN, SIZE, DURATION:**

This retrospective, single-center study included a cohort of 1429 couples who started their IVF treatment at a tertiary center between January 2014 and April 2021 as the development dataset. An additional 613 couples who started treatment at the same center between April 2021 and December 2023 were used as the temporal validation dataset.

**PARTICIPANTS/MATERIALS, SETTING, METHODS:**

Predictive performance was assessed using the following metrics: prediction error (Brier score, scaled Brier score), time-dependent AUC (cumulative/dynamic AUC, mean AUC, and time-dependent precision-recall AUC), and calibration (mean calibration, calibration plot, and calibration slope). Both pre-treatment and post-treatment models predicted the cumulative live birth success rates of up to six complete IVF cycles. Various discrete time survival analysis methods were trained, evaluated, and compared with the baseline models. Discrete time survival analysis models include the ones embedded with a generalized linear model (Logit) and tree-based ensembles (Bayesian Additive Regression Trees (BART), random forests). The baseline models are the refitted/recalibrated pre-treatment and post-treatment McLernon models, considering factors available in our data.

**MAIN RESULTS AND THE ROLE OF CHANCE:**

The discrete time survival analysis models, especially BART, performed better on our data than the baseline models especially at the post-treatment phase. Validation results showed moderate discriminative ability for the pre-treatment model, with a mean AUC 0.69 (95% CI: 0.65, 0.73) and a scaled integrated Brier score (IBS) of 0.13 (13% performance improvement over Kaplan–Meier estimates). For the post-treatment model, discriminative power was strong, with a mean AUC of 0.82 (95% CI: 0.79, 0.85) and a scaled IBS of 0.19. In the BART model, the most important feature was female age and embryo utilization rate (EUR) in the pre- and post-treatment models, respectively.

**LARGE SCALE DATA:**

N/A

**LIMITATIONS, REASONS FOR CAUTION:**

Our study is limited by the retrospective and single-center design, including moderate sample size and long observation time span. Methodologically, several features were not present in our data when refitting the McLernon baseline models.

**WIDER IMPLICATIONS OF THE FINDINGS:**

This work illustrates how discrete time survival analysis ML models can be used to generate individualized, multi-cycle IVF prognoses. These ML models can be readily trained on local populations, which facilitates center-specific predictions. Our findings support ongoing efforts to improve patient counseling through data-driven communication about the individualized treatment plans.

**FUNDING:**

This study was supported by grants from the national Research Foundation—Flanders (FWO) (personal mandate 1235924N to F.K.N., research grant G046024N to C.V., T.T.S.) and the Flemish Government (Flanders AI Research program).

**DISCLOSURES:**

The funders had no role in the study design, data collection or analysis, publication decision or manuscript preparation. There are no conflicts of interest to be declared.

WHAT DOES THIS MEAN FOR PATIENTS? Infertility is a multi-factorial disease and often requires IVF treatment. IVF treatment can be physically and emotionally demanding, and most patients might need several attempts before success. This uncertainty makes it hard for doctors to help patients shape long-term and realistic expectations. Reliable outcome predictions can enable doctors to help patients feel more informed and supported throughout their treatment.

We developed data-driven models using machine learning, a type of artificial intelligence, to estimate the probability of having a baby following each IVF attempt and across multiple tries. These models used information such as age and treatment details to give personalized estimates. We tested the models on real patient data and found that they were more accurate than existing methods.

Models can be used as a counseling aid to help doctors communicate realistic chances of success in a data-supported way, alongside their clinical experience. This can make counseling more transparent and reduce uncertainty. While the models were developed using data from a single fertility center, they have the potential to be evaluated and adapted for use in other clinics in future studies.

## Introduction

Infertility is a global health issue affecting one in every six people of reproductive age worldwide, which often requires IVF treatment (Mburu *et al.*, 2023). In Belgium, despite substantial financial support for up to six cycles of IVF treatment from the government, high dropout rates and treatment discontinuation remain persistent clinical challenges. Approximately half of couples drop out after experiencing two failed cycles (De Neubourg *et al.*, 2021). Though the underlying causes are complex, these persistent challenges are partially attributable to the misaligned expectations and the communication gap between patients and health care providers. Patients tend to hold overly optimistic expectations (Devroe *et al.*, 2023; McMahon *et al.*, 2024), and the high medical knowledge barrier can make it difficult to convey realistic outcomes (Yao *et al.*, 2024a). Fortunately, accurate prognostic predictive models can assist as counselling tools, making communication more effective and straightforward before and during IVF treatment (Brew *et al.*, 2023; Yao *et al.*, 2024a) and have been reported to be associated with increased IVF utilization rates (Wang *et al.*, 2022). Historically, IVF outcome prediction models have predominantly focused on short-term outcomes, such as success over one embryo transfer or one treatment cycle. However, long-term prognosis obtained from survival analysis is more informative, as it provides dynamic risk estimates over time and helps clinicians tailor personalized treatment plans to prevent both over- and under-treatment (Malchau *et al.*, 2017; Harrison *et al.*, 2021). A systematic review by Ratna *et al.* (2020), which examined IVF prediction models developed between 1978 and 2019, identified 23 studies. Most of these models demonstrated moderate discriminative power, with C-statistics ranging from 0.6 to 0.7, and no artificial intelligence (AI)/machine learning (ML) methods were identified.

In recent years, however, ML methods have emerged as a promising approach (Yao *et al.*, 2024b, 2025), demonstrating remarkable improvements in the predictive performance (Sadegh-Zadeh *et al.*, 2024). In particular, ensemble tree-based models showed superior discriminative power (Qiu *et al.*, 2019; Goyal *et al.*, 2020; La Marca *et al.*, 2021; Liu *et al.*, 2023; Li *et al.*, 2025), with the best post-treatment model achieving an area under the receiver operating characteristic curve (ROC AUC) above 0.8 (Liu *et al.*, 2023). However, these studies continued to focus primarily on binary classification tasks (e.g. success or not over one cycle) and failed to model cumulative success rates or account for the longitudinal nature of the IVF treatment (multiple cycles) (Yland *et al.*, 2019).

To our best knowledge, only two previous studies made predictions over multiple cycles (Stolwijk *et al.*, 2000; McLernon *et al.*, 2016). Concretely, Stolwijk *et al.* (2000) employed a discretized proportional hazards model under the assumption of continuous time distribution, but the discriminative performance was not reported. The model developed by McLernon *et al.* (2016), which was later updated using a US national dataset (McLernon *et al.*, 2022), pioneered the application of discrete time survival analysis to predict the cumulative live birth rate (CLBR) for IVF patients. Discrete time survival analysis has been recommended in IVF settings due to its ability to handle the unique characteristics of fertility treatment data, including the discrete nature of treatment cycles and the time-varying probability of success (Maity *et al.*, 2014). In addition, this framework allows greater flexibility in the choice of predictive models while appropriately accounting for censoring (Tutz and Schmid, 2016).

The McLernon models are well-recognized in this domain and represent an important methodological advance in IVF prognosis. Supported by the large national registry data, these models adopted a discrete time survival analysis framework with logistic regression, using features transformed with cubic splines to capture non-linear effects. Their development has substantially improved predictive performance, with C-statistics increasing from above 0.60 to above 0.70 (Ratna *et al.*, 2020), which is a strong baseline.

Importantly, the McLernon models leverage large-scale national registry-based data to ensure broad applicability across clinical settings. However, such registry-based models are constrained by the clinical and laboratory features available in the registry. For instance, McLernon *et al.* (2022) included only the number of oocytes as a post-treatment-specific feature.

The complementary aspect of developing center-specific models allows incorporation of more clinical and laboratory features. For example, embryo utilization rate (EUR) has been shown to be positively associated with the live birth rate (Datta *et al.*, 2025). Similarly, the endometrial thickness is an important prognostic factor (Holden *et al.*, 2018; Mahutte *et al.*, 2022). However, these two factors have often been overlooked in IVF predictive models (Ratna *et al.*, 2020; McLernon and Bhattacharya, 2023).

In this study, we leverage the strength of discrete time survival analysis and ML models, as one of the first studies (i) to improve multi-cycle prognostic estimates (ii) to explore the prognostic value of EUR and endometrial thickness.

## Materials and methods

### Data sources

This study was approved by the KU Leuven University Privacy and Ethics Platform (G-2024-8905) and the University Hospitals Leuven (UZ Leuven) Ethics Committee (S69890). As a retrospective study, no specific informed consent was requested, but consent for using medical data for quality management purposes was granted by the patients. The data were extracted from UZ Leuven fertility patient records and pseudonymized. This manuscript complies with the TRIPOD-AI checklist Version 11—January 2024 (Collins *et al.*, 2024).

### Study population

IVF cycle treatment information was collected from UZ Leuven between January 2014 and December 2024. Data included couples who commenced IVF treatment using their own eggs and sperm between January 2014 and December 2023, ensuring that each couple had at least 1 year of treatment history. Couples who started IVF treatment between January 2014 and April 2021 were included in the development dataset, while those who started between April 2021 and December 2023 were assigned to the temporal validation dataset.

The exclusion criteria included:

1. Women outside the reimbursement-eligible age range (18–43 years).
2. Couples undergoing treatment involving oocyte, sperm, or embryo donation, or a gestational carrier.
3. Couples with embryo transfers using banked embryos resulting from fertility preservation.
4. Couples who have undergone pre-implantation genetic testing (PGT), including PGT for aneuploidy (PGT-A) and PGT for monogenic disorders (PGT-M).
5. Couples who received IVF treatment before January 2014 at UZ Leuven or other clinics.

Details of the data preprocessing steps and excluded couples are illustrated in Supplementary Fig. S1.

### Baseline characteristics

In the pre-treatment model, the baseline characteristics included female and male age, female and male BMI, infertility duration, baseline FSH, serum anti-Müllerian hormone (AMH), number of female pathologies, infertility type, and diagnostic factors, including ovulation problems (yes vs no), tubal problems (yes vs no), implantation problems (yes vs no), severe endometriosis (yes vs no), and male pathology (yes vs no). The number of female pathologies was calculated as the sum of diagnoses across four categories: ovulatory disorders, endometriosis, tubal pathology, and implantation problems (each coded as 1 for yes and 0 for no). Infertility type was classified based on pregnancy history: women with any prior pregnancy, regardless of outcome or gestational stage, were classified as having secondary infertility, while those without prior pregnancies were classified as having primary infertility. Severe endometriosis was defined as having an American Fertility Society (AFS) score ≥16 (Stage III). Male pathology was diagnosed based on WHO criteria (World Health Organization, 2021). Baseline FSH and AMH were measured at Day 3 of the menstrual cycle.

In the post-treatment model, six additional post-treatment-specific features were included: the last measured endometrial thickness before oocyte aspiration (EM), the number of days between EM last measurement and oocyte aspiration, the number of retrieved oocytes, the number of two-pronuclear (2PN) zygotes, the presence of a morula on Day 3 of embryo development, and the EUR. EUR is defined as the proportion of usable embryos, calculated with the sum of the embryos freshly transferred and frozen divided by the number of 2PN zygotes (ESHRE Special Interest Group of Embryology and Alpha Scientists in Reproductive Medicine, 2017). For couples with no 2PN zygotes available, EUR is set to zero. EUR ranges from 0 to 1 ([0, 1]).

Features used to refit the baseline McLernon model included female age, BMI, AMH, ovulation problems, and male pathology in the pre-treatment phase; and female age, BMI, number of oocytes retrieved, ovulation problems, and male pathology in the post-treatment phase. The features used in the original McLernon model but absent from our dataset were (i) four categorical features in the pre-treatment model: pregnancy history (whether having full-term birth (≥37 weeks)), polycystic ovarian syndrome, uterine abnormalities, and unexplained infertility; (ii) two diagnostic features in the post-treatment model, including polycystic ovarian syndrome and uterine abnormalities.

Table 1 summarizes the baseline characteristics of the development and validation datasets.

**Table 1.** Feature characteristics for the development and validation datasets

Metrics		Development dataset	Temporal validation dataset
Number of couples		1429	613

**Pre-treatment features**			
Female age		33.06 ± 4.73	33.77 ± 4.53
Male age		36.19 ± 6.61	36.81 ± 6.50
Female BMI		23.91 ± 4.19	24.21 ± 4.59
	Missing	42 (2.9%)	52 (8.5%)
Male BMI		26.07 ± 3.93	26.31 ± 4.23
	Missing	555 (38.8%)	279 (45.5%)
Infertility duration		3.24 ± 2.32	3.59 ± 2.74
	Missing	135 (9.4%)	309 (50.4%)
number of pathology		0.98 ± 0.96	0.88 ± 0.90
FSH		7.40 ± 3.31	8.26 ± 6.21
	Missing	580 (40.6%)	401 (65.4%)
AMH		3.30 ± 3.56	3.05 ± 2.55
	Missing	547 (38.3%)	314 (51.2%)
Cause of infertility			
Infertility type (secondary)		518.0 (36.45%)	219.0 (36.5%)
	Missing	8 (0.6%)	13 (2.1%)
Ovulation problem (yes)		453.0 (32.33%)	164.0 (27.29%)
	Missing	28 (2%)	12 (2.0%)
Tubal problem (yes)		346.0 (36.15%)	112.0 (27.45%)
	Missing		205 (33.4%)
Implantation problem (yes)		171.0 (12.84%)	78.0 (13.64%)
	Missing	97 (6.8%)	41 (6.7%)
Severe endometriosis (yes)		216 (15.12%)	91 (14.85%)
Male pathology (yes)		819.0 (57.31%)	281.0 (46.6%)
	Missing		10 (1.6%)

**Post-treatment specific features**			
Endometrial thickness		9.49 ± 2.25	9.23 ± 2.31
	Missing	6 (0.4)	
Days to endometrial thickness measure		2.42 ± 0.78	2.56 ± 1.04
	Missing	6 (0.4%)	
Embryo utilization rate		0.57 ± 0.33	0.51 ± 0.32
number of 2PN zygotes		4.91 ± 4.03	5.56 ± 4.70
number of oocytes retrieved		8.81 ± 6.12	9.80 ± 6.73
	Missing	1 (0.1%)	
Morula Day 3 (yes)		27 (1.89%)	22 (3.59%)

AMH, serum anti-Müllerian hormone.

### Outcome

The time unit used in this study is the treatment cycle. A complete cycle includes all fresh and frozen embryo transfers resulting from the same hormonal stimulation episode (Wilkinson *et al.*, 2021). Canceled cycles due to treatment complications or no eggs or embryos available are also considered as a complete cycle (Wilkinson *et al.*, 2021; McLernon *et al.*, 2022). The maximum observation time is six cycles, and the study follows couples until a cycle that results in the first live birth or until the last complete cycle that does not lead to a live birth. Couples who failed but did not complete six cycles of treatment or succeeded after more than six cycles are considered censored.

Both the pre-treatment and post-treatment models predict the CLBR over a maximum of six complete IVF cycles. A live birth is defined as a delivery of at least one viable newborn after a minimum gestational age of 24 weeks, marking the end of one IVF treatment episode. Only the first live birth was considered in this study; live births resulting from the subsequent treatment episodes were excluded (no recurrent events).

### ML models

Discrete time survival analysis ML models include the ones that are embedded in generalized linear model logit link (Logit) with elastic net regularization and in tree-based ensembles: Bayesian Additive Regression Trees (BART) (Chipman *et al.*, 2010) and random forests (RF) (Breiman, 2001).

Discrete time survival analysis treats event time on a discrete scale and models conditional hazards within specific intervals (Tutz and Schmid, 2016). The survival function for subject i at time t is derived by the conditional risk $S_{i}(t|X_{i})=\prod_{j:t_{j}\leq t}1-\lambda_{ij}(X_{i})$ with $\lambda_{ij}$ as the conditional hazards at each interval before time t.

Usually, in survival analysis, outcome is expressed with pairs of event time t and event indicator δ (1 indicates experiencing the event; 0 indicates right censoring), as illustrated in Fig. 1, left panel. In the current setting, the time component is a cycle, which is intrinsically discrete (Scheike and Jensen, 1997).

**Figure 1.**
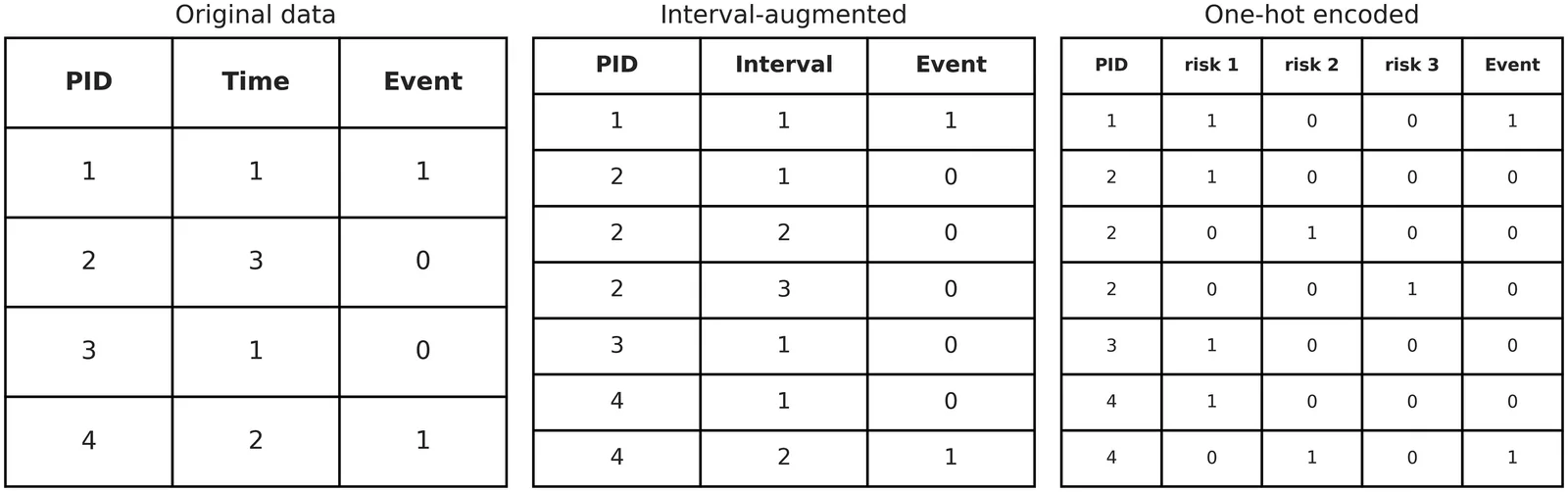
**Data augmentation examples.** Left (original data): one row is one patient (PID) with observed time and event indicator (1 = event, 0 = censored). Middle (Interval augmented): the example data are expanded to person-cycle format. One row is one patient per discrete time interval at risk; event is the outcome indicator to each interval. Right (One hot encoded): each interval is represented by a set of mutually exclusive risk indicators (risk 1–3).

In both Logit and RF models, the dataset was preprocessed for the discrete time survival analysis through the Python package survstack (https://github.com/kaboroevich/survstack.git), which reshapes the dataset with one-hot-encoded interval indicators (Fig. 1, right panel). The Logit model was penalized with both L1 and L2 norm (elasticnet). RF is an ensemble tree method that uses both bootstrap sampling and random feature selection per split (Breiman, 2001).

Five-fold cross-validation was used for hyperparameter tuning (search space and best values can be found in Supplementary Table S1). In the Logit model, the inverse of regularization strength (C), and the weight distribution between L1 and L2 penalty (l1_ratio) were optimized to prevent overfitting. In RF, the number of trees was set to 100. Hyperparameter tuning was applied to the maximum depth of the tree (max_depth) to prevent over-complex trees, the number of features to be considered when deciding the best split (max_features) to improve generalization, the minimum number of samples required for further split (min_samples_split), and the minimum number of samples at each leaf (min_samples_leaf) to reduce overfitting.

Since data were augmented and one patient can correspond to multiple rows to different time intervals, 5-fold cross validation was stratified on the patient level to prevent data leakage across person-cycle rows (a single patient is exclusively included in either training folds or the held-out fold).

BART is a Bayesian nonparametric method that uses a collection of trees to approximate f(x)=E(Y|x) (Chipman *et al.*, 2010). Similar to the gradient boosting method, each tree in BART acts as a weak learner and is fitted iteratively to the residuals of the ensemble. The weak learner is enforced by a regularization prior, and the iterative fitting is performed using Bayesian backfitting to iteratively make changes on a fixed number of trees. In BART, feature importance does not need to be additionally computed, and it can be represented with the relative frequency of each feature chosen in the splits. Additionally, under the Bayesian paradigm, BART provides the uncertainty of the predictions.

The BART models were set up with 1000 iterations as burn-in, 1000 returned posterior draws, and base parameter 0.95. Hyperparameter tuning was applied to the power parameter (power), the number of trees (m), the number of prior SD (k), and whether to perform variable selection or not (sparse), with 5-fold cross validation in which the splits were stratified on the event status (Supplementary Table S1). Explanation of the hyperparameters can be found in Supplementary File S1 (Bayesian Additive Regression Trees section). Bayesian optimization was used to search for the best parameters (Snoek *et al.*, 2012). Both pre-treatment and post-treatment BART models provide individual prediction uncertainties through 95% quantile bands from 1000 posterior draws, meaning that there is a 95% probability that the true survival probability lies within the specified range.

Dataset preprocessing for the BART model was implemented with the BART package using the surv.pre.bart function, which treats time as a parametric time feature (Fig. 1, middle panel) (Sparapani *et al*., 2016).

#### Packages

Logit and RF modeling, along with hyperparameter tuning, were implemented using the scikit-learn library (version 1.7.2) in Python (Pedregosa *et al.*, 2011). BART modeling was implemented using the BART package (version 2.9.9) in R (Sparapani *et al.*, 2016), and hyperparameter optimization was performed using the rBayesianOptimization package (version 1.2.1) in R (Yan, 2025).

### Statistical analysis

#### Missing data

In the pre-treatment model, 9/14 features and 10/14 features had missing values in the development and validation dataset, respectively. FSH suffered most from missing values, with 40.6% missingness in the development set. For post-treatment-specific features, only a few cases had missing values in the development dataset, and no missing values were detected in the validation dataset. The detailed information about missing frequencies and percentages can be seen in Table 1.

To impute the missing values, we employed the MissForest algorithm (Stekhoven and Bühlmann, 2012), which utilizes a chained RF approach, where features with the lowest number of missing values are imputed first. RF regressors and RF classifiers were used to impute continuous and categorical features, respectively. In both pre-treatment and post-treatment models, we fit an imputation model on the development data and then applied it to impute missing values in the validation data. After imputation, the numerical features were scaled using SD computed from the development dataset and applied to the validation dataset for both pre-treatment and post-treatment datasets (Cheng *et al.*, 2023). We applied the same SD scaling for consistency across models, as it was required for the regularized linear models, even though it was not necessary for tree-based models.

#### Evaluation metrics

Metrics used to evaluate predictive performance include scaled Brier score, cumulative/dynamic AUC, mean AUC, time-dependent precision-recall AUC (PR-AUC), mean calibration, and calibration plots.

The scaled Brier score is used to evaluate model accuracy and performance improvement in comparison with the Kaplan–Meier estimates (or referred to null model with no features, non-parametric statistic) (Kattan and Gerds, 2018). The scaled Brier score is defined as $1-\frac{\mathrm{Brier}\;\mathrm{score}\;\mathrm{from}\;\mathrm{chosen}\;\mathrm{model}}{\mathrm{Brier}\;\mathrm{score}\;\mathrm{from}\;\mathrm{Null}\;\mathrm{model}}$. Two different scaled Brier scores are derived depending on the Brier score: (i) when the Brier score is used as the time-dependent Brier score, the scaled Brier score is the *time-dependent scaled Brier score*; (ii) when the Brier score is used as the integrated Brier score (IBS), the scaled Brier score is the *scaled IBS*. Time-dependent Brier score is the mean-squared error between the true survival status and the predicted survival probabilities at time point t, weighted by the inverse probability of censoring weights (IPCW). IBS is the average Brier score over a specified time interval. In our case, IBS is the average Brier score over five cycles. Though predictions are made up to the sixth cycle, the Brier score at the last time point (sixth cycle) cannot be computed due to the unknown censoring distribution (unknown censoring weights).

Since a lower Brier score indicates more accurate model performance, a higher scaled Brier score indicates higher model performance improvement. A scaled Brier score of zero indicates that model performance is the same as the Kaplan–Meier estimates, and a score of one indicates a perfectly accurate model.

Cumulative/dynamic AUC and mean AUC are discriminative measures. Cumulative/dynamic AUC is used to quantify how well a model can distinguish the participants who encounter the event by time t from the ones who encounter the event after time t. Higher values mean higher discriminative power of the model. Cumulative/dynamic AUC at a specific time t is derived from a cumulative true positive rate (TPR) and dynamic false positive rate (FPR) and is weighted with IPCW. Mean AUC is the average time-dependent cumulative/dynamic AUC over five cycles, instead of six cycles, same reason as the Brier score. In addition, we included time-dependent area under precision recall curve (PR-AUC) as complementary metrics to cumulative/dynamic AUC (Beyene *et al.*, 2024). Time-dependent PR-AUC evaluates the model’s ability to correctly identify positive cases, particularly when negative cases dominate. We employed two available methods to compute PR-AUC, namely Yuan method (Yuan *et al.*, 2018) and Beyene method (Beyene *et al.*, 2024). Detailed methodological descriptions and explanations can be found in the original papers.

We estimated 95% CI for the scaled Brier score, integrated scaled Brier score, cumulative/dynamic AUC, mean AUC, and time-dependent PR-AUC using nonparametric bootstrapping on 1000 resamples of the validation dataset, recalculating the evaluation metrics in each replicate and taking the 95% bounds across bootstrap distributions.

Mean calibration (also called calibration-in-the-large) measures the agreement between the predicted and observed survival fraction (McLernon *et al.*, 2023). At time point t, mean calibration is the ratio of mean observed event faction and the average predicted risk (Observed/expected, O/E). Due to the presence of censoring in the data, the observed survival fraction is estimated with the Kaplan–Meier estimator. An O/E ratio of one with narrow CI indicates better calibration performance.

A calibration plot also provides a visual assessment of the agreement between the observed and predicted CLBR. It is constructed by dividing the range of predicted CLBR into 10 bins, each containing an equal number of patients. The plot then compares the Kaplan–Meier estimates against the average predicted risks within each bin. Calibration curve is not a quantitative measure, but a linear fit of the data with a slope closer to one, an intercept closer to zero, and narrower CI means a better calibration performance.

The library scikit-survival (version 0.25.0) in Python was employed to compute time-dependent Brier score, IBS, cumulative/dynamic, and mean AUC (Pölsterl, 2020). Time-dependent PR-AUC was computed in the library APTools (version 6.8.8) for Yuan method (Cai *et al.*, 2018), and library tdPRC (version 1.0.0) for Beyene method (Beyene *et al.*, 2025).

#### Partial dependence plot

A partial dependence plot (PDP) visualizes the marginal effects of one or two features on the prediction of a model by fixing the chosen feature values (Goldstein *et al.*, 2015). A PDP can reveal the complex relationships between features and targets.

#### Baseline model

We refitted/recalibrated McLernon models (McLernon *et al.*, 2022) using the overlapping features in both pre-treatment and post-treatment models, and the observation time was extended to six cycles because (i) different feature sets were present in our dataset; (ii) the observation time in the original McLernon models was up to three cycles and the predictions were made only from the second to third cycle in the post-treatment model. The refitted McLernon models used the same transformed features as the original ones, modeled through cubic splines. We updated transformed feature coefficients and the intercepts to capture cycle-to-cycle associations. The refitted McLernon formulas can be found in Supplementary File S1 (Refitted McLernon Model section).

## Results

Table 1 shows the distributions of the features in the development and validation datasets, as well as the missingness. The number of patients in the development and validation datasets is 1429 and 613, respectively. Mean (SD) observation time in days is 2758.7 (SD 786.5) and 847.5 (SD 287.1); mean (SD) observation time in cycles is 2.0 (SD 1.3) and 1.7 (SD 1.1) in development and validation dataset, respectively. The percentage of patients that participate in Cycles 1–6 is 47.9, 25.1, 13.1, 7.1, 3.6, and 3.2% in the development set; 57.1, 24.4, 10.0, 5.9, 1.6, and 1.0% in the validation set, respectively. Fourteen and 20 features were included in the pre-treatment and post-treatment model, respectively (Devroe *et al.*, 2020; Ratna *et al.*, 2020; McLernon *et al.*, 2022; Xia *et al.*, 2024). As shown in Supplementary Table S2, 848/1429 (59.3%) and 373/613 (60.8%) patients had a live birth within six cycles in the development and validation dataset, respectively. The number of women who had a live birth, did not have a live birth but continued the treatment, and did not have a live birth and discontinued the treatment across six IVF cycles is shown in Supplementary Table S2. Fig. 2 presents the average CLBR across six cycles, estimated with 1 - Kaplan–Meier estimator (cumulative incidence curve). It shows that the validation dataset had a significantly better prognosis for the first two cycles. More concretely, the CLBR after the first and second cycle was 33.9% (95% CI 31.5, 36.4%) and 52.6% (49.9, 52.6%) in the development dataset, and 45.02% (41.18, 49.05%) and 59.2% (55.1, 63.4%) in the validation dataset. After stratifying on female age, similar results were observed (Supplementary Fig. S2).

**Figure 2.**
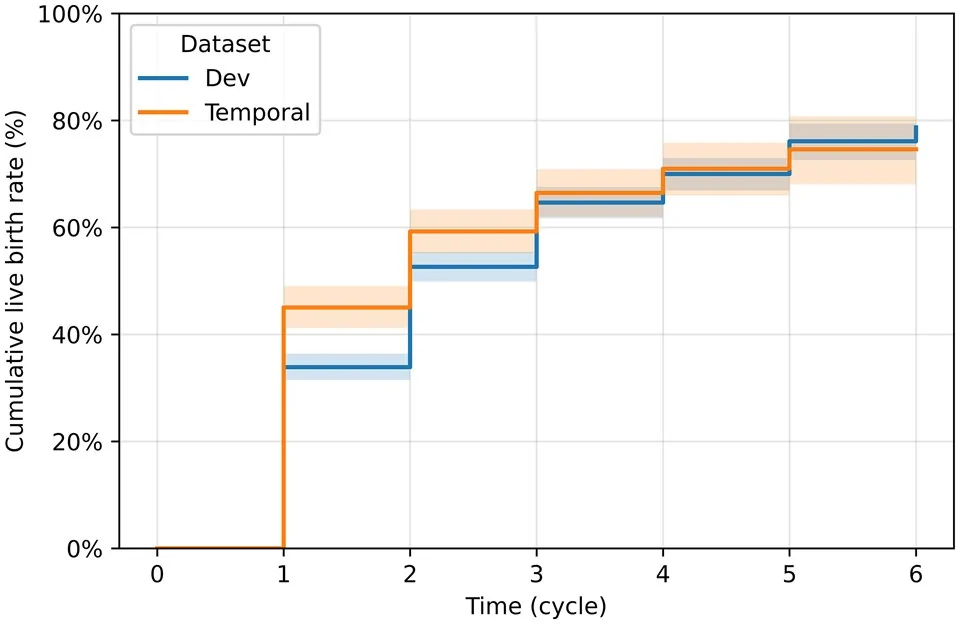
**1 - Kaplan–Meier curves with 95% CI for CLBR according to the total number of cycles needed for a live birth.** CLBR, cumulative live birth rate.

### Pre-treatment model

The pre-treatment model utilizes information at the diagnostic stage (Table 1). The mean (SD) maternal age at the beginning of IVF treatment was 33.06 (SD 4.73) years in the development dataset and 33.77 (4.53) years in the validation dataset. The mean infertility duration was 3.24 (2.32) and 3.59 (2.74) years in the developmental and validation datasets, respectively; 57.31 and 46.6% of patients had male pathology in the development and validation dataset, respectively.

As shown in Table 2, among pre-treatment ML models, BART had the best performance with the highest performance improvement over the Kaplan–Meier estimates with scaled IBS 0.13 (95% CI 0.07, 0.19) and the highest discrimination power with mean AUC 0.69 (0.65, 0.73). Figure 3 illustrates the evolution of scaled Brier score and cumulative/dynamic AUC score across five cycles. BART had a stable increase in discriminative performance, with AUC above 0.7 after the first cycle (Supplementary Table S3).

**Figure 3.**
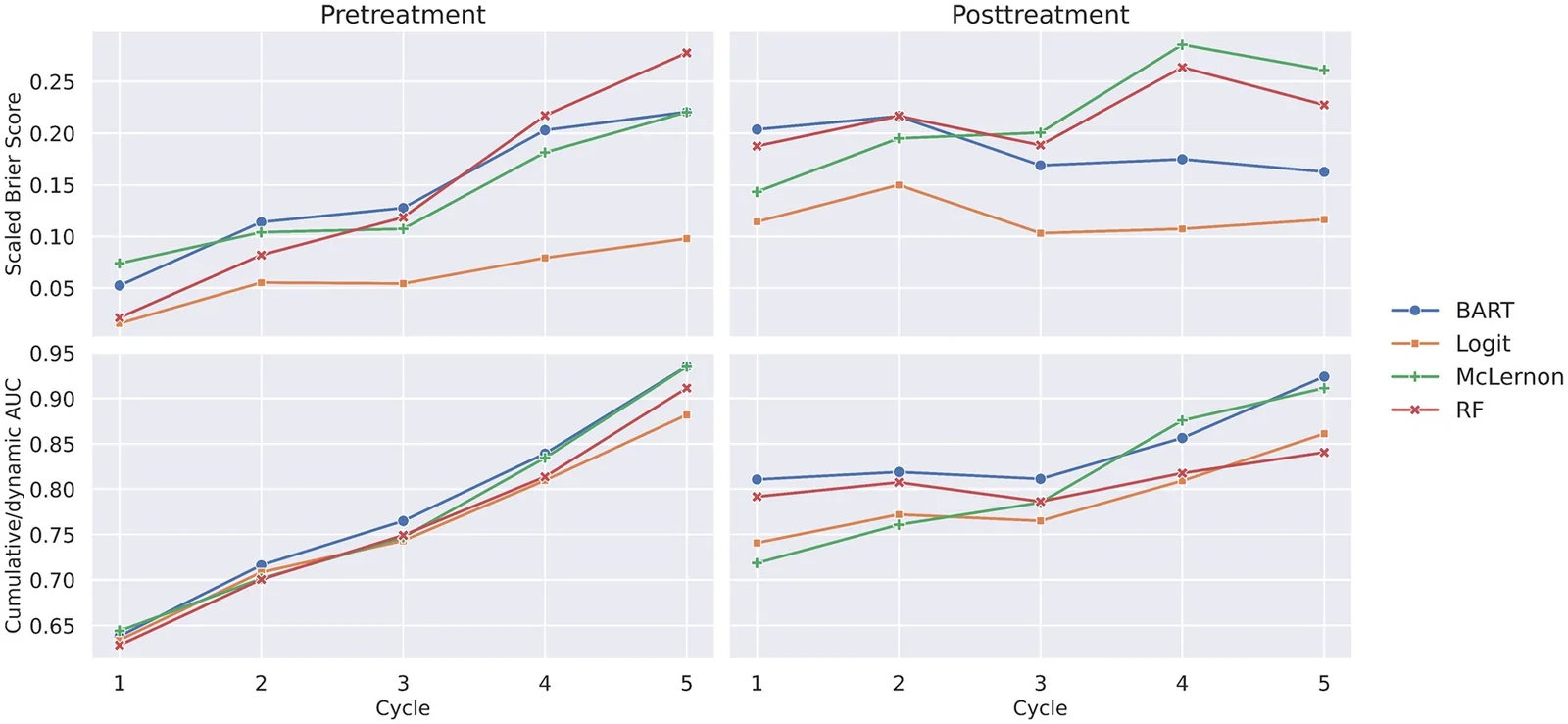
**Time-dependent validation performance.** Left column: pre-treatment models; right column: post-treatment models. Top row: time-dependent scaled Brier score; bottom row: time-dependent cumulative/dynamic AUC. *X*-axis: cycle number; *Y*-axis: performance values. Though the predictions were made up to sixth cycle, censoring distribution was unknown at sixth cycle, so performance can only be computed up to fifth cycle. AUC, area under the curve; BART, Bayesian additive regression trees; RF, random forest.

**Table 2.** Performance metrics for both pre-treatment and post-treatment models over six cycles

	Pre-treatment		Post-treatment	
Model	scaled IBS	mean AUC	scaled IBS	mean AUC
BART	**0.13 (0.07, 0.19)**	**0.69 (0.65, 0.73)**	0.19 (0.12, 0.26)	**0.82 (0.79, 0.85)**
RF	0.11 (0.07, 0.16)	0.68 (0.64, 0.72)	**0.21 (0.15, 0.27)**	0.80 (0.76, 0.83)
Logit	0.05 (0.00, 0.10)	0.68 (0.64, 0.72)	0.12 (0.05, 0.19)	0.76 (0.72, 0.79)
McLernon	0.11 (0.03, 0.17)	0.68 (0.64, 0.72)	0.19 (0.13, 0.25)	0.74 (0.70, 0.78)

BART, Bayesian additive regression trees; RF, random forest; IBS, integrated Brier score; AUC, area under curve. Bold values indicate the best-performing model.

The refitted McLernon baseline model had a scaled IBS value of 0.11 (0.03, 0.17) and a mean AUC value of 0.68 (0.64, 0.72). Though the BART model had comparable overall performance with McLernon model, at the first cycle when positive cases were fewer (positive rate 33.9%), BART was better at identifying the positive cases with time-dependent PR-AUC 0.64 (0.57, 0.71) and 0.56 (0.51, 0.62), compared with McLernon model with time-dependent PR-AUC 0.61 (0.54, 0.67) and 0.53 (0.48, 0.60) in Yuan and Beyene methods, respectively.

Figure 3 illustrates the evolution of scaled Brier score and cumulative/dynamic AUC score across five cycles. BART had a stable increase in discriminative performance, with AUC above 0.7 after the first cycle.

The calibration plot (Fig. 4) demonstrates the overall calibration performance across treatment cycles. It is evident that for the first two cycles, all models underestimated the predicted CLBR, with the O/E ratio significantly above one. However, at Cycles 4 and 5, both BART and refitted McLernon models achieved nearly perfect calibration, with only minor deviations from an O/E ratio of one.

**Figure 4.**
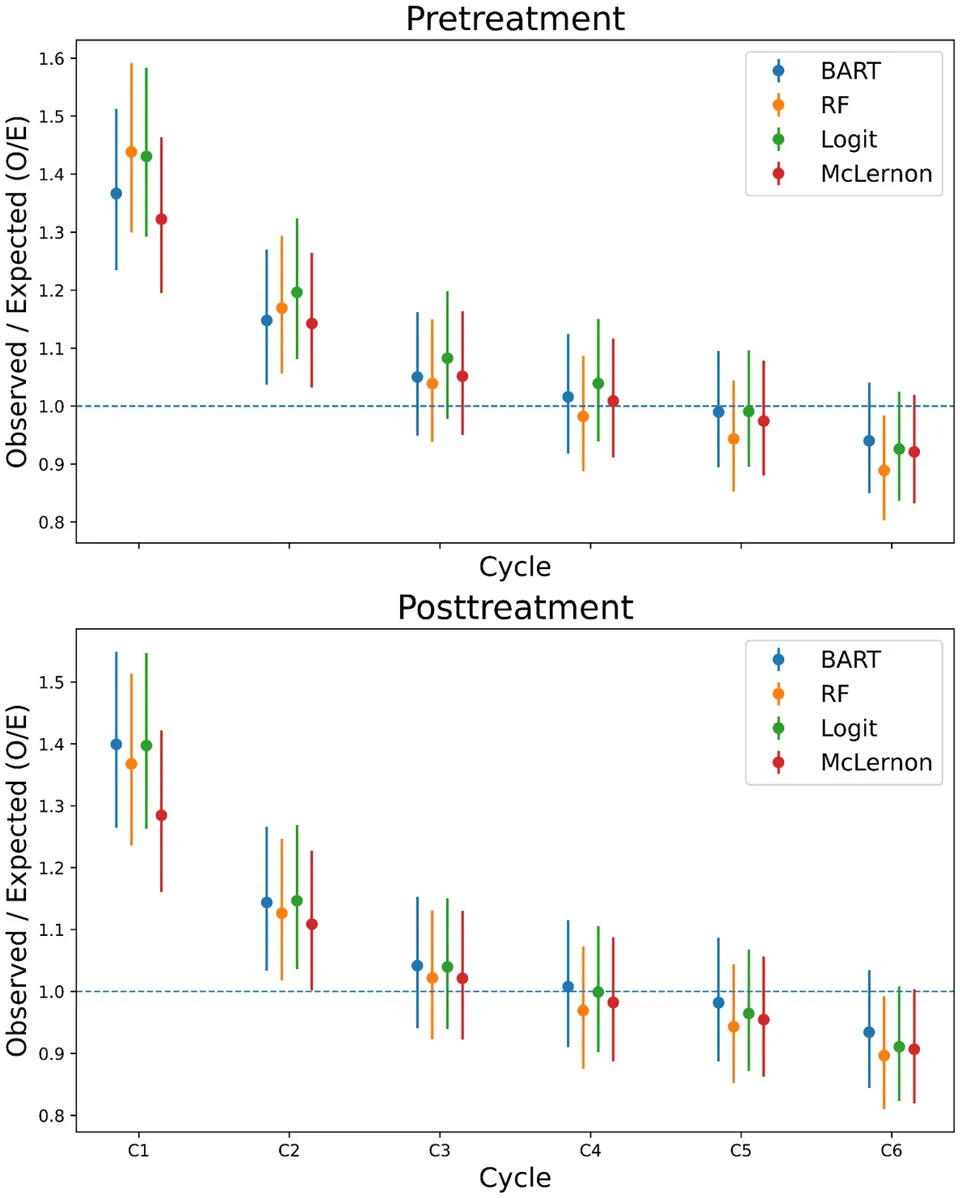
**Mean calibration for each cycle.** Top row: pre-treatment models; bottom row: post-treatment models. Observed: Kaplan–Meier estimates from validation dataset; Expected: mean predictions for each cycle. *X*-axis: cycle number; *Y*-axis: ratio values. BART, Bayesian additive regression trees; RF, random forest.

Calibration performance was primarily assessed with the calibration slope. The BART model demonstrated the best calibration performance compared with the other ML methods by having a slope 1.28 (95% CI 1.11, 1.45), 1.18 (0.99, 1.38), 1.00 (0.83, 1.17), 1.07 (0.74, 1.39), 1.13, (0.76, 1.49), and 1.15 (0.77, 1.53) across six cycles (Fig. 5; Supplementary Figs S3 and S4 for RF and Logit model). For example, although RF showed competitive performance with BART, its calibration was consistently poorer in all time points with slope being 1.37 (0.78, 1.96), 1.33 (0.97, 1.69), 1.16 (0.82, 1.51), 1.44 (1.11, 1.78), 1.54 (1.21, 1.86), and 1.6 (1.27, 2.01).

**Figure 5.**
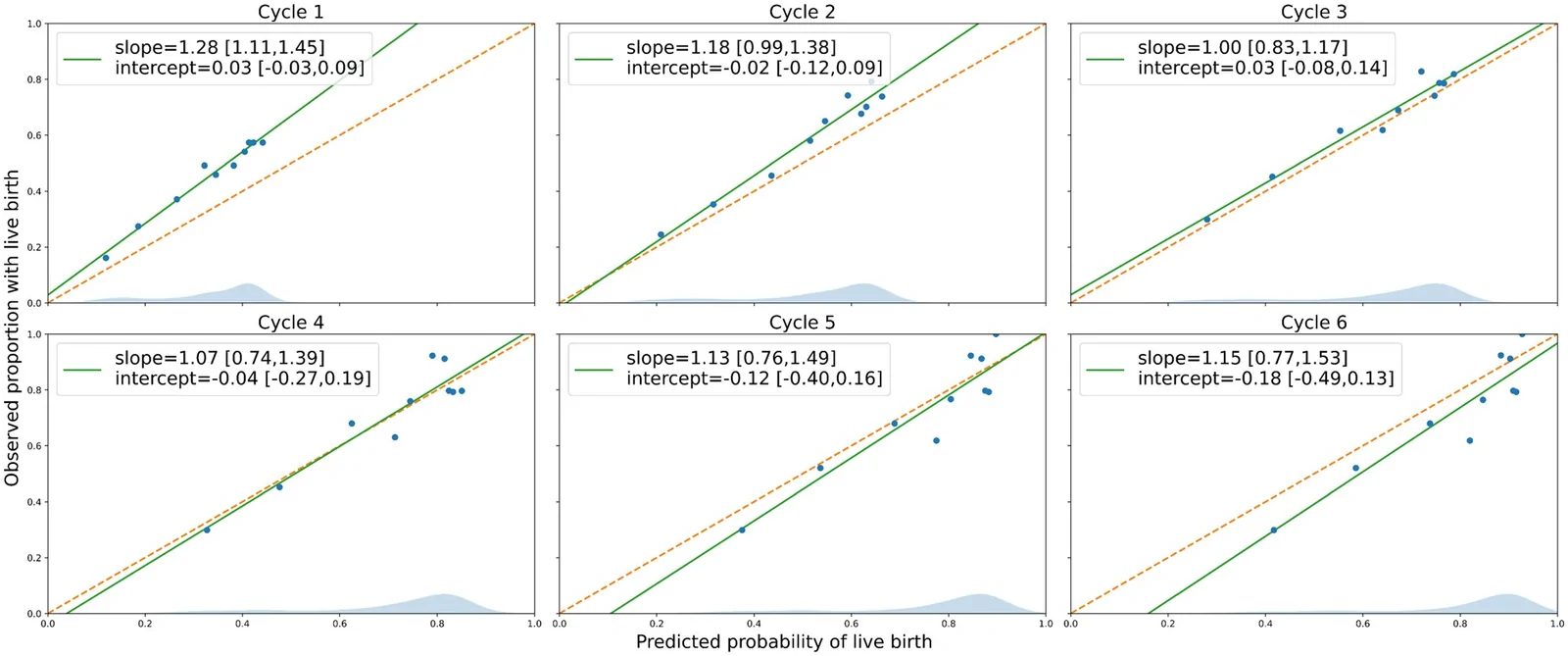
**Calibration plots by cycle for the pre-treatment BART model**. For each cycle, *X*-axis: the mean predicted cumulative probability of live birth within each bin; *Y*-axis: the observed cumulative proportion of live birth in the validation dataset within each bin. The density plot shows the distribution of predictions and the dashed line represents perfect calibration. The 10 points were from 10 bins, which were obtained by ranking all patients in the validation dataset by their predicted probabilities and then dividing them into 10 equally sized groups. BART, Bayesian additive regression trees.

The refitted McLernon baseline model was better calibrated than BART (calibration slope: 1.09 (0.82, 1.36), 0.96 (0.73, 1.20), 0.82 (0.58, 1.07), 0.94 (0.52, 1.36), 0.99 (0.60, 1.38), and 1.02 (0.63, 1.41)), as shown in Supplementary Fig. S5.

### Post-treatment model

The post-treatment model incorporated the post-treatment-specific features such as EM, EUR, the number of oocytes retrieved, and the total number of 2PN zygotes (Table 1). In the development and validation datasets, the mean (SD) EM was 9.49 (2.25) and 9.23 (2.31) mm, respectively. The EUR was 0.57 (0.33) and 0.51 (0.32); the number of oocytes retrieved was 8.81 (6.12) and 9.80 (6.73); and the total number of 2PN zygotes was 4.91 (4.03) and 5.56 (4.70), respectively.

Post-treatment performance is summarized in Table 2. Among all the models, BART achieved the highest discriminative performance with a mean AUC value of 0.82 (95% CI 0.79, 0.85), significantly outperforming the refitted McLernon baseline model (mean AUC 0.75 (0.71, 0.79)). The RF model ranked second in the discriminative ability (mean AUC 0.80 (0.76, 0.83)) and had the highest performance improvement with a scaled IBS of 0.21 (0.15, 0.27).

Time-dependent AUC and scaled Brier scores are shown in Fig. 3. BART presented strong discrimination with cumulative/dynamic AUC above 0.8 across six cycles (Supplementary Table S4). Additionally, BART showed high performance improvement with scaled Brier scores: 0.2 (0.16, 0.24) and 0.22 (0.15, 0.28) at the first two cycles, but decreased at Cycle 3 (0.17 (0.08, 0.25) and then plateaued (Supplementary Table S4). The higher scaled IBS of the RF was mostly attributed to the higher performance gains in the last two cycles compared to BART (Supplementary Table S4). Moreover, time-dependent PR-AUC results (Supplementary Table S5) illustrated that the BART post-treatment model performed better in discriminating the positive cases at the first two cycles. The BART model achieved time-dependent PR-AUC values of 0.81 (0.75, 0.87) and 0.71 (0.65, 0.77) using Yuan and Beyene methods, respectively, at the first cycle, which increased to 0.89 (0.83, 0.94) and 0.81 (0.76, 0.85) at the second cycle. In contrast, the McLernon model showed lower performance with time-dependent PR-AUC values of 0.68 (0.61, 0.75) and 0.60 (0.54, 0.66) at the first cycle; 0.86 (0.79, 0.93) and 0.75 (0.70, 0.81) at the second cycle. Worth mentioning, since positive cases became more dominant in later cycles, the higher time-dependent PR-AUC in both models does not necessarily mean better performances.

Similar to the pre-treatment model, all models underestimated CLBR at the first two cycles (mean calibration plot, Fig. 4). BART models were well-calibrated at Cycles 4 and 5, and the calibration plot showed similar results where CLBR was underestimated at Cycle 1 with the slope value being 1.31 (95% CI 0.97, 1.65) and intercept being 0.03 (−0.10, 0.15) (Fig. 6). Calibration plots for other models can be found in the Supplementary Figs S6, S7, and S8.

**Figure 6.**
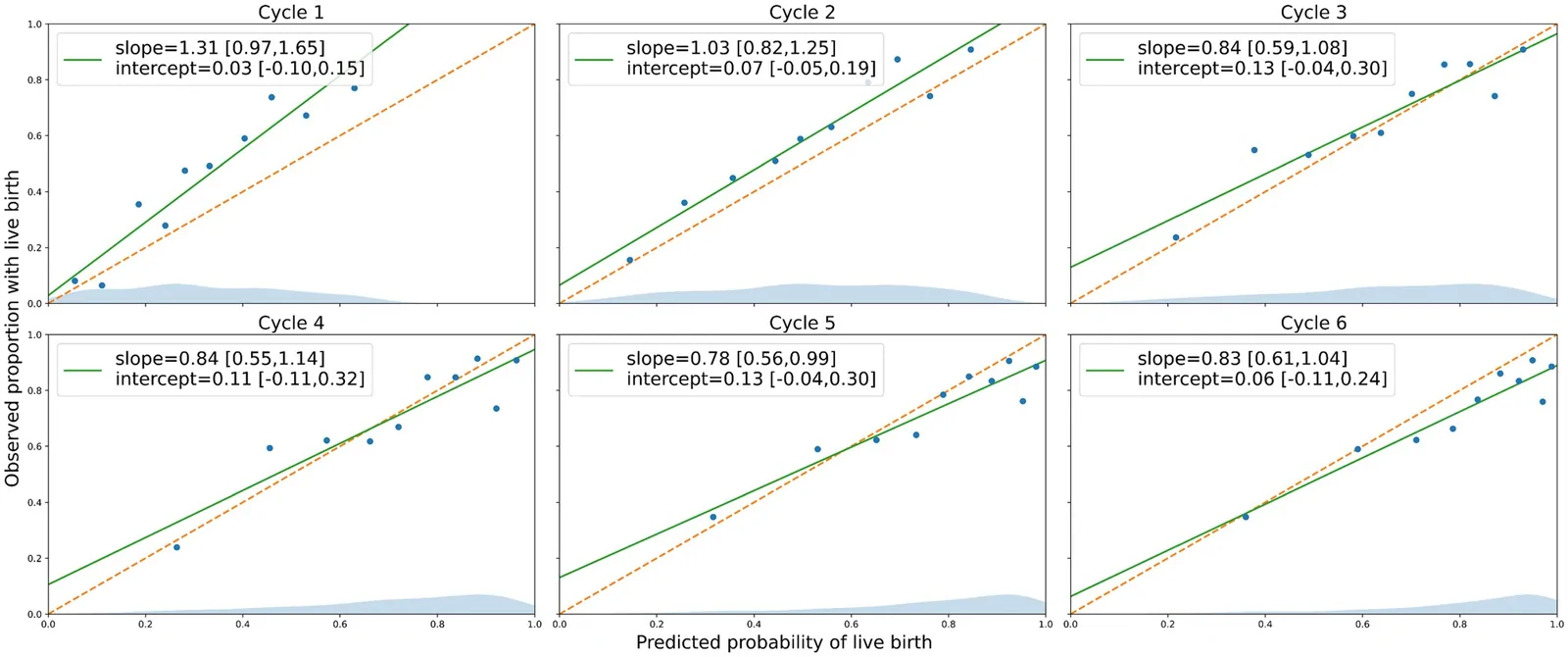
**Calibration plots by cycle for the post-treatment BART model.** For each cycle, *X*-axis: the mean predicted cumulative probability of live birth within each bin; *Y*-axis: the observed cumulative proportion of live birth in the validation dataset within each bin. The density plot shows the distribution of predictions and the dashed line represents perfect calibration. The 10 points were from 10 bins, which were obtained by ranking all patients in the validation dataset by their predicted probabilities and then dividing them into 10 equally sized groups. BART, Bayesian additive regression trees.

### Prognostic exploratory analysis

In the pre-treatment BART model, the couple with the lowest predicted prognosis has an estimated CLBR of 7% at Cycle 1, rising to 28% by Cycle 6; this couple did not achieve a live birth and discontinued the treatment after the first cycle. In contrast, the couple with the most favorable prognosis had an estimated CLBR of 46% at Cycle 1 and 94% by Cycle 6, and achieved a live birth in the first cycle. In the post-treatment BART model, the couple with the lowest predicted prognosis had an estimated CLBR of 1% at Cycle 1, increasing to 14% by Cycle 6 and did not achieve a live birth after four cycles; the couple with the best predicted prognosis had a CLBR of 71% at Cycle 1 and had a live birth in the first cycle.

Since reimbursement up to three cycles is more internationally applied, the performance metrics over the first three cycles are included in Table 3. Results were broadly consistent with those obtained over six cycles. BART had the best discrimination in both pre- and post-treatment models with mean AUC of 0.71 (0.66, 0.75) and 0.81 (0.78, 0.85), respectively.

**Table 3.** Performance metrics for both pre-treatment and post-treatment models over the first three cycles.

	Pre-treatment		Post-treatment	
Model	Scaled IBS	Mean AUC	Scaled IBS	Mean AUC
BART	**0.10 (0.06, 0.14)**	**0.71 (0.66, 0.75)**	**0.20 (0.15, 0.26)**	**0.81 (0.78, 0.85)**
RF	0.07 (0.03, 0.11)	0.69 (0.64, 0.74)	0.20 (0.15, 0.25)	0.80 (0.75, 0.83)
Logit	0.04 (0.00, 0.08)	0.70 (0.65, 0.74)	0.13 (0.08, 0.18)	0.76 (0.72, 0.80)
McLernon	0.10 (0.05, 0.15)	0.70 (0.65, 0.75)	0.18 (0.13, 0.23)	0.75 (0.71, 0.80)

BART, Bayesian additive regression trees; RF, random forest; IBS, integrated Brier score; AUC, area under curve. Bold values indicate the best-performing model.

In addition, we performed an exploratory subgroup analysis focusing on couples with the poorest predicted outcomes (Table 4). In the pre-treatment model, the mean predicted three-cycle CLBR was lower among couples who did not achieve a live birth within three cycles than in the overall validation cohort: 0.57 (SD 0.19) versus 0.68 (SD 0.13). Among the five couples whose predicted three-cycle CLBR fell below 20% (mean predicted CLBR: 18%), none achieved a live birth, although the small sample size limits interpretation.

**Table 4.** Observed and predicted cumulative live birth rates for patients below selected thresholds.

Model	Threshold	n	Events	Mean predicted CLBR	Observed CLBR
Pre-treatment	<20%	5	0	0.18	0
	<30%	42	10	0.25	0.24
Post-treatment	<10%	3	0	0.08	0
	<20%	21	1	0.14	0.05
	<30%	59	11	0.21	0.19

Threshold, patients with cumulative success rate over three cycles smaller than certain cutoff; n, the number of patients within the group; events, the number of patients with live birth; CLBR, cumulative live birth rates over three cycles.

In the post-treatment model, the corresponding mean predicted three-cycle CLBR was similarly lower among couples who did not achieve a live birth than the ones in the overall cohort (0.54 (SD 0.23) versus 0.64 (SD 0.22)). The post-treatment model identified a substantially larger subgroup with poorest prognoses: among the 21 couples with a predicted three-cycle CLBR below 20% (mean predicted CLBR: 14%), only one ultimately achieved a live birth.

### Feature importance and PDPs

The most important features in the pre-treatment BART model were female age, cycle number, and infertility duration, while the most important features in the post-treatment model were EUR, cycle number, female age, and the number of 2PN zygotes (Fig. 7).

**Figure 7.**
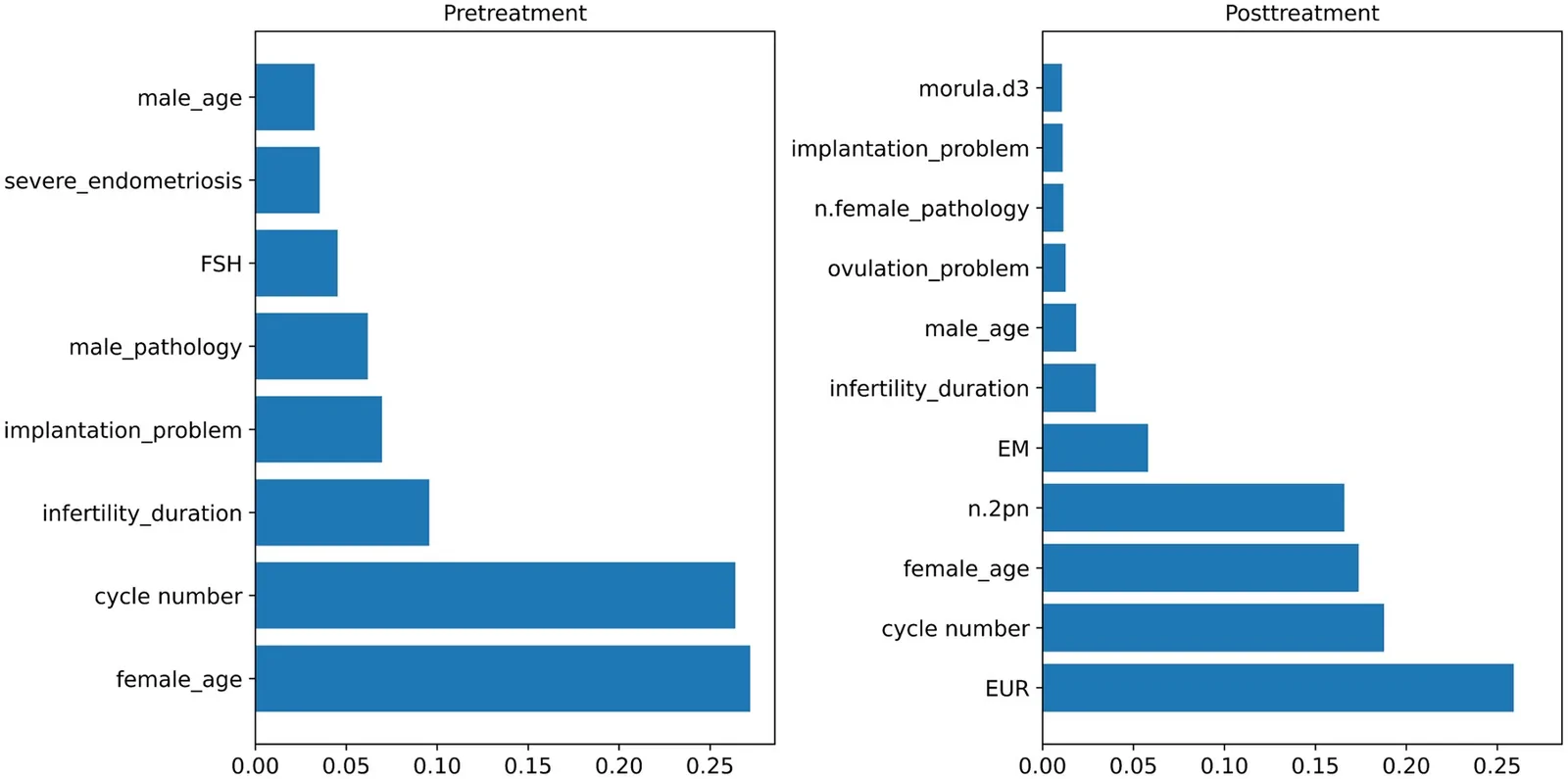
**Feature importance for pre- and post-treatment models.** Left column: the pre-treatment BART model; right column: the post-treatment BART model. *X*-axis: the normalized importance for each predictor in the BART model for CLBR; *Y*-axis: Top 7 and Top 10 important features for pre- and post-treatment models, respectively. Larger values indicate a greater contribution to the predictions. EM, endometrial thickness; 2pn: embryos with two pronuclei; EUR, embryo utilization rate; BART, Bayesian additive regression trees; CLBR, cumulative live birth rate.

To explore the relationships between key features and outcomes, we generated PDPs for the top four features for both models (Figs. 8 and 9). In the pre-treatment model (Fig. 8), the partial dependence functions revealed a nonlinear relationship between female age and survival probability (the probability of not achieving live birth). Increasing female age was not associated with worse prognosis until age was above 20–30. Additionally, the age-related negative effects on the prognosis were more pronounced between 35 and 40 compared to 30–35. In contrast, for infertility duration, ovulation problem, and implantation problem, the PDPs showed minimal differences, if any, across different feature values, suggesting that nearly no independent predictive value was detected in the pre-treatment context.

**Figure 8.**
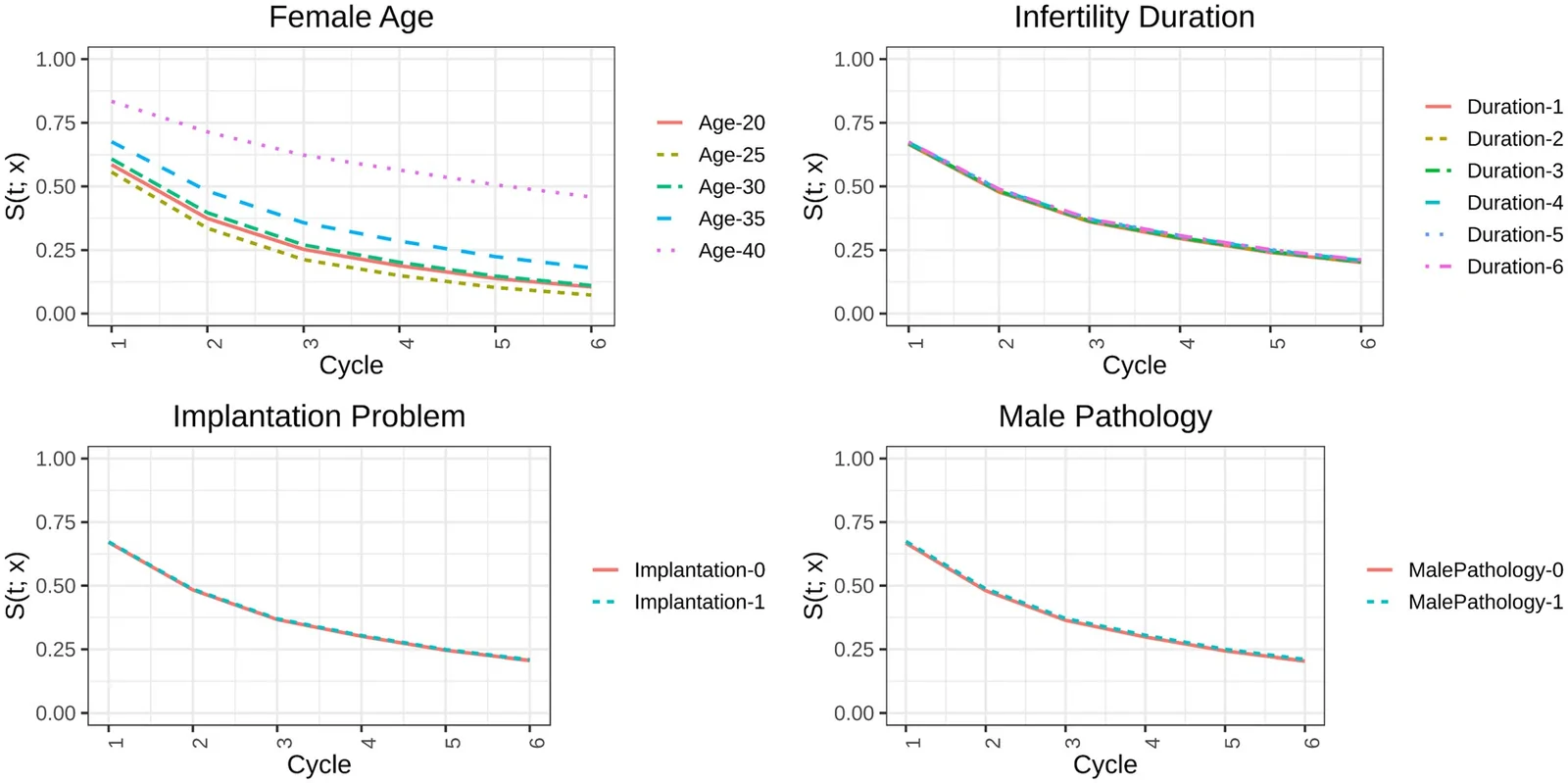
**Partial dependence plots for top four features from the pre-treatment model.** Each panel shows the BART survival function (not having live birth over nth cycle) across IVF cycles for different values of a single predictor, while keeping all other features original. At each panel, the *X*-axis represents cycle number and the *Y*-axis the predicted probability of not yet having achieved a live birth by that cycle. BART, Bayesian additive regression trees.

**Figure 9.**
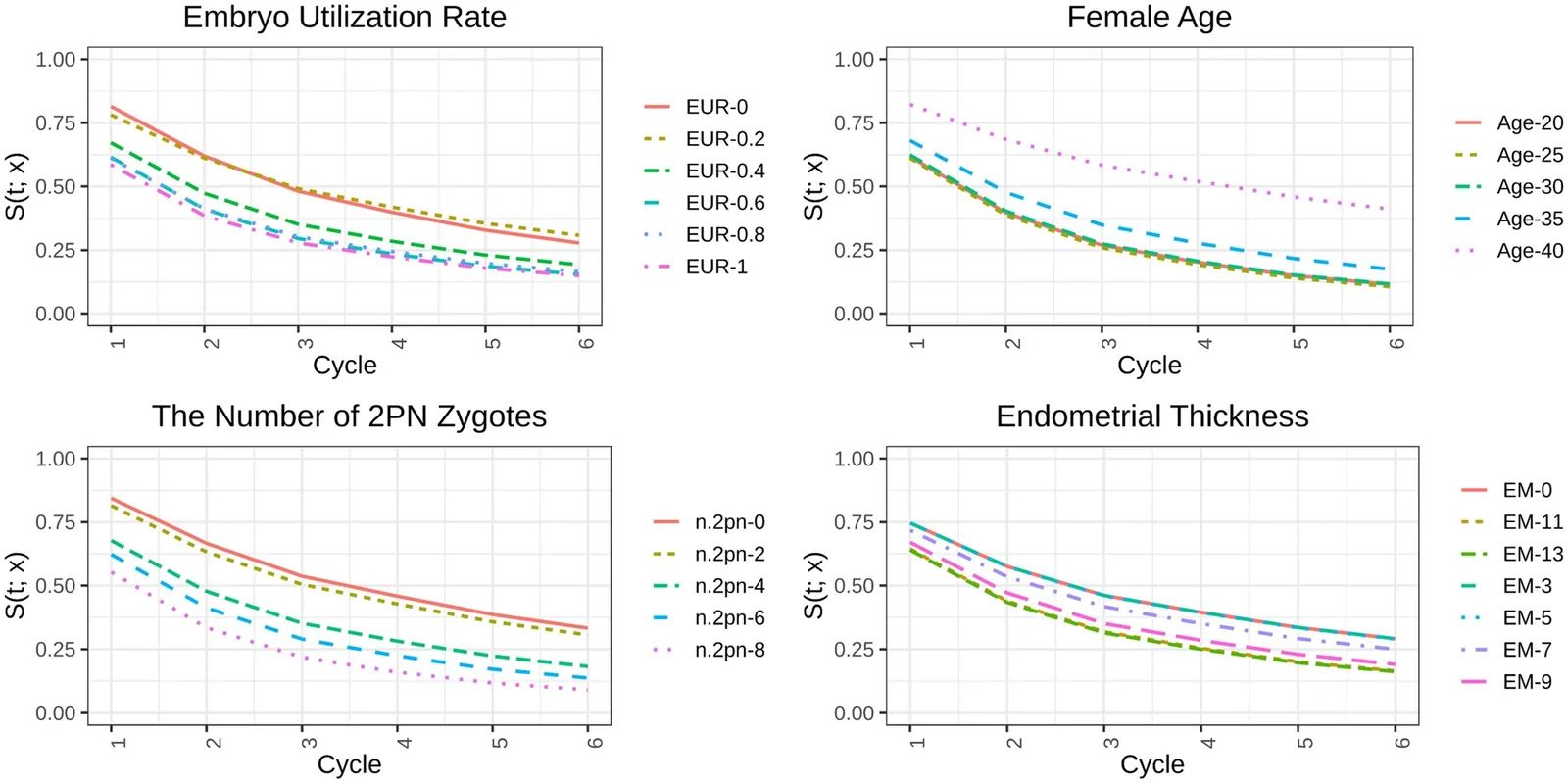
**Partial dependence plots for top four features from the post-treatment model.** Each panel shows the BART survival function across IVF cycles for different values of a single predictor, while keeping all other features original. At each panel, the *X*-axis represents cycle number and the *Y*-axis the predicted probability of not yet having achieved a live birth by that cycle. 2pn, embryos with two pronuclei; BART, Bayesian additive regression trees.

The post-treatment model revealed nuanced relationships between the top four features and outcomes (Fig. 9). EUR demonstrated the strongest effect on the prognosis as the top feature. The best prognosis (highest probability of achieving live birth) was observed when EUR was between 0.6 and 1. EUR increasing from 0.2 to 0.4 led to the most rapid improvement of the IVF outcome (survival curve declined rapidly), and the poorest prognosis was observed when EUR values were equal to 0 or 0.2.

In the post-treatment model, survival curves remained nearly the same from age 20 to 30, after which age-related differences started to show, and more advanced age had an adverse effect on the prognosis.

For the number of 2PN zygotes, the PDP suggested better prognosis with an increasing number of 2PN zygotes within the range of 0 to 8. The PDP for EM showed that within the range 5–11 mm, the thicker endometrium was associated with better prognosis. However, when thinner than 5 mm or thicker than 11 mm (11–13 mm), the effect of EM on the prognosis diminished.

The 2D PDPs (Fig. 10) demonstrated the interaction between age and the number of 2PN zygotes, and the interaction between age and EUR. With increasing female age, the higher number of 2PN zygotes (above 4) had a larger impact on the IVF prognosis. However, through the interaction between age and EUR, relative prognoses remain nearly the same at different EUR groups, with only age effects being pronounced, especially with more advanced age.

**Figure 10.**
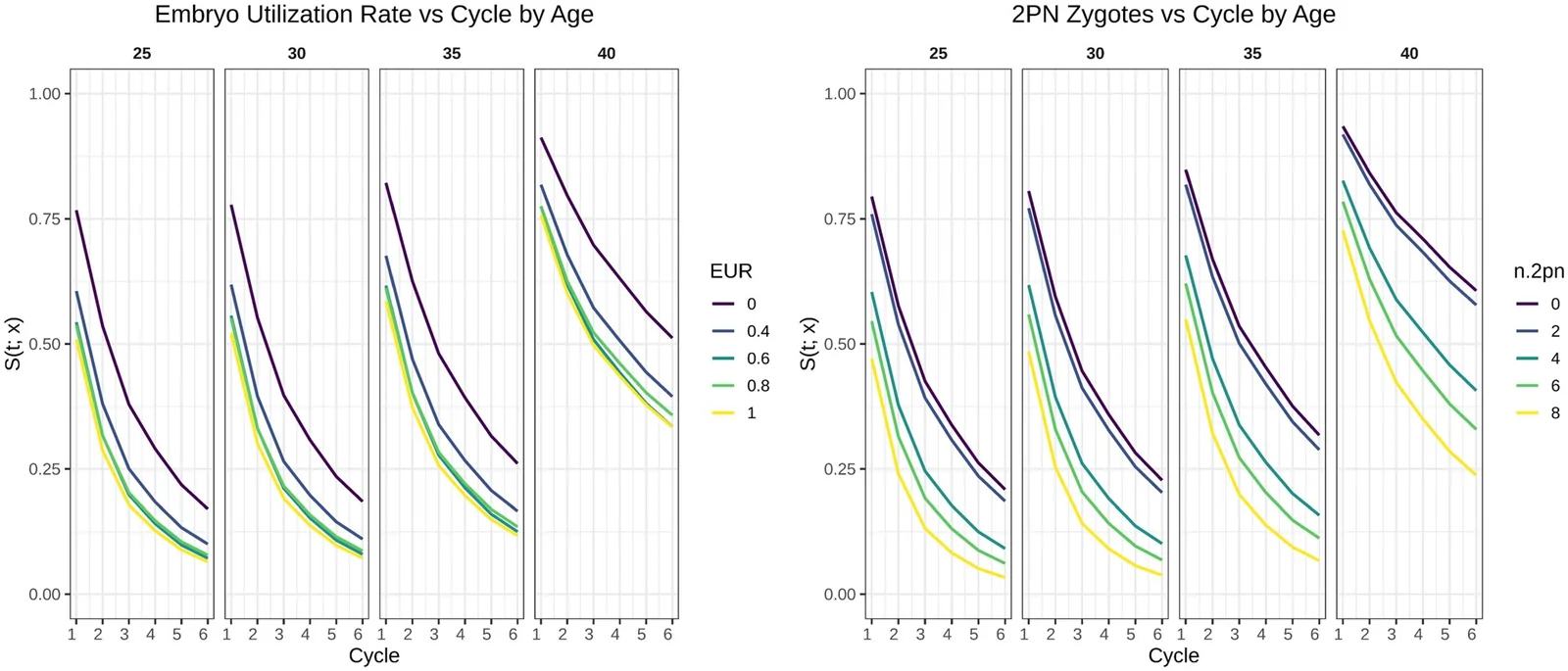
**Two-dimensional dependence plots from the post-treatment BART model.** Each panel shows the BART survival function across IVF cycles for different values of two predictors, while keeping all other features original. Left panel: combinations of EUR and female age; right panel: combinations of the number of 2pn zygotes and female age. 2pn, embryos with two pronuclei; BART, Bayesian additive regression trees; EUR, embryo utilization rate.

## Discussion

### Main findings

Few studies have reported IVF survival analysis outcome estimates, and their predictive performances have generally been moderate for both pre-treatment and post-treatment models (Stolwijk *et al.*, 2000; McLernon *et al.*, 2016, 2022). To our best knowledge, we were one of the first studies (i) to use discrete time survival analysis ML models to predict CLBR and achieved significantly improved performance, from above 0.7 to above 0.8 of the C-statistics in the post-treatment model; (ii) to demonstrate the important prognostic value of EUR and EM, and confirm the non-linear effects of them to the outcome.

The best-performing model, BART, achieved moderate discrimination in the pre-treatment phase (mean AUC: 0.69 (95% CI: 0.65, 0.73) over six cycles; 0.71 (0.66, 0.75) over three cycles) and strong discrimination in the post-treatment phase (mean AUC: (0.82 (95% CI: 0.79, 0.85) over six cycles; 0.81 (0.78, 0.85) over three cycles)). The post-treatment model has the potential of identifying a subgroup with the poorest prognoses. In the pre-treatment BART model, the most important predictor was female age. In the post-treatment BART model, key predictors included EUR, EM, female age, and the number of 2PN zygotes.

### Strengths

Our study demonstrated several key strengths. First, we employed discrete time survival analysis to properly handle censoring and survival outcomes, together with ML methods to improve the prediction outcome. We not only achieved comparable performance in the pre-treatment model but also demonstrated a substantial improvement in the post-treatment model when compared with the refitted McLernon baseline models.

Second, longitudinal predictions by survival analysis offer richer information than binary classification tasks (D’Hondt *et al*., 2026), with overall outcome evolution and dynamic outcomes at each time point. For example, as illustrated by an example in Supplementary File S1 (Use case section), Supplementary Table S6, and Supplementary Fig. S9, the BART survival model generates both cumulative and conditional probabilities together with the quantification of prediction uncertainties, which bring more clinical insights. More concretely, the decreasing success rate at each cycle combined with the increasing CLBR addresses both the challenges from repeated cycles and the benefits of treatment persistence, supporting informed, transparent, and more realistic counseling.

Third, our models, especially the post-treatment model, demonstrated the potential of identifying clinically meaningful subgroup of couples with particularly poor prognosis. Couples identified in this subgroup might require more careful counseling, including early discussion of treatment regimen, and the emotional preparation with low probability of success. Though ambitious, accurate prognostic estimates could ultimately support the personalization of the reimbursement strategies and improve the efficiency of healthcare resource allocation.

Fourth, the tree-ensemble survival models such as BART efficiently captured the non-linearity between the features and the outcome and demonstrated the importance of EUR and EM as key prognostic factors. We use female age, EUR, the number of 2PN zygotes, and EM as examples to further elaborate this. In the pre-treatment model, female age emerged as the most important feature. Consistent with the previous study (McLernon *et al.*, 2022), the optimal age was ∼20–30 years old when the best prognosis was observed. In both pre-treatment and post-treatment models, ages beyond 35 witnessed a rapid decline in the prognosis, which was widely observed in previous studies (Heazell *et al.*, 2018; Zhang *et al.*, 2021; Liu *et al.*, 2023). In the post-treatment model, we found that EUR, which reflects overall embryo quality, was positively associated with a live birth when its value was below 0.6, which mildly agrees with a recent study (Datta *et al.*, 2025). This association was consistent among women aged 25–40 years, suggesting that within this age range, the age-related decline in prognosis may be at least partly mitigated by higher EUR. In addition, between ages 35 and 40, the prognosis was increasingly dominated by maternal age, as evidenced by the systematic worsening of the prognosis, while the relative prognostic differences between different EUR levels remained relatively stable. Also, patients with no usable embryos (EUR of value 0) in the first treatment cycle consistently have the worst prognosis in all age groups. While this observation should be interpreted cautiously, it highlights a potentially important early signal of limited reproductive potential.

The number of 2PN zygotes has also been understudied in previous predictive models (Commenges-Ducos *et al.*, 1998; Carrera-Rotllan *et al.*, 2007). In our study, embryo count emerged as an important predictor: a higher number of 2PN zygotes was associated with better pregnancy outcomes, and this association became more pronounced as the number increased. Moreover, the positive effect of the number of 2PN zygotes was stronger at more advanced maternal ages, suggesting that older women may benefit more from having a larger number of 2PN zygotes.

Successful pregnancy requires viable embryos, a receptive endometrium, and the synchrony between them (Blanco-Breindel *et al.*, 2025). EM is routinely evaluated in the fertility centers and has been shown to be an independent predictor of IVF outcomes (Zhao *et al.*, 2012; Yuan *et al.*, 2016; Holden *et al.*, 2018). In our model, we identified a non-linear association between EM and pregnancy outcomes, showing significantly better prognosis for patients with EM >11 mm compared to those with <7 mm, consistent with previous findings (Holden *et al.*, 2018). In addition, in contrast to EUR, which may be more sensitive to culture conditions and affected by the inter- and intra-observer variation, EM is a relatively objective measure, more comparable across different clinical centers.

Fifth, this study demonstrates the complementarity of developing single-center models to developing registry-based models. Our study confirms the value of using survival analysis, including post-treatment laboratory data in a non-linear way (McLernon *et al.*, 2022) and the added value of including clinical data such as endometrial thickness. These learnings can be used by clinicians and researchers from other clinics interested in developing their own models to estimate highly accurate, personalized multiple-cycle prognosis for their patients.

Lastly, we used the TRIPOD-AI checklist to prepare the manuscript, ensuring transparent definition of predictors, handling of the missing data, and model evaluations. Code is also made publicly available for reference.

### Weaknesses and research implications

This study is limited by its moderate sample size over a 10-year study period. Though this long timeframe was chosen to include a larger number of eligible patients, it may have introduced temporal heterogeneity in clinical practice, leading to suboptimal model calibration, especially at the first two cycles.

These calibration discrepancies are likely due to the temporal changes in clinical practice and laboratory procedures at the study center. In particular, these changes include the introduction of new incubation systems facilitating low-oxygen culture conditions (Wale and Gardner, 2010), the adoption of a state-of-the-art clean room environment, and the transition into blastocyst culture. These changes may have contributed to the improvements in the implantation outcomes over time and may partly explain the higher observed live birth probabilities in the temporal validation cohort compared with the development cohort. The observed distribution shift also highlights the importance of model monitoring during deployment, even in a single-center setting. In particular, model discrimination and calibration should be periodically re-assessed in new patients, and potential shifts in predictor and outcome distributions should be routinely monitored.

Worth mentioning, at the sixth cycle, calibration performance presented a tendency toward overestimation. One possible reason is that, although we ensured all patients had at least 1 year of IVF treatment (Stolwijk *et al.*, 2000; McLernon *et al.*, 2022), patients enrolled in more recent years inevitably had shorter observed calendar time, which may lead to outcome bias, particularly in later cycles.

In addition, the implementation of the McLernon model in our study was compromised by data availability. We were only able to include 6/9 features used in the original McLernon pre-treatment model and 5/7 features in the post-treatment model, which may lead to the suboptimal performance of the McLernon model. Nevertheless, the Mclernon model was the most relevant external comparator, utilizing discrete survival analysis for predictions over multiple IVF cycles.

Moreover, our pre-treatment model did not include the antral follicle count, an important indicator of ovarian reserve function (Grande *et al.*, 2014). We were unable to extract this feature due to technical difficulties, and the absence of this feature probably is one of the reasons for the less satisfying performance of the pretreatment model. Also, the diagnosis of ‘unexplained infertility’ was absent in our study, which is one of the most commonly used features in previous studies (Ratna *et al.*, 2020). This absence does not necessarily reflect a true lack of such patients but is largely attributable to how diagnoses are recorded in the fertility center. First, ‘unexplained infertility’ is an exclusion diagnosis and therefore is often not actively entered into patient records. Second, in UZ Leuven, as a tertiary center, additional diagnostic tests are often performed later in the treatment trajectory. As a result, patients may initially meet criteria for unexplained infertility but subsequently receive a more specific diagnosis, resulting in the absence of this diagnosis in our dataset. Third, couples who are diagnosed with unexplained infertility are more likely to have PGT-A procedure and were excluded from our dataset.

Our single-centric setting only allowed temporal validation, and no external validation on other IVF clinics was demonstrated. However, contemporary perspectives on external validity emphasize that it is not limited to a fixed fitted model but rather the transferability of mechanisms, predictive relationships, and methodological strategies across settings (Findley *et al.*, 2021).

Furthermore, features used in this study were static from a certain phase of the treatment; thus their longitudinal nature was underexplored. Future work can focus on developing dynamic prediction models that update risk estimates after each IVF cycle by incorporating the latest information (e.g. age, embryo characteristics from each cycle of the treatment). For example, updating age at each cycle would reflect the impact of treatment discontinuation/interruption on IVF outcomes (Harrison *et al.*, 2021, 2022).

Considering that post-treatment models surpassed their pre-treatment counterparts, we may regard the first cycle as more diagnostically informative. That is, couples should first undergo the first cycle, before being informed about their prognosis. Now that the model performance has improved significantly, multiple stakeholders including statisticians, clinicians, patient representatives, and policy makers should join force to decide on the sufficient level of model performance and calibration in order for personalized estimates to be communicated to patients, or even to inform reimbursement decisions. More research should challenge the findings of our group, investigate whether communicating personalized prognosis improves realistic expectations without causing anxiety (Devroe *et al.*, 2022), and study its impact on treatment discontinuation.

## Conclusion

We employed discrete time survival analysis ML models to predict long-term IVF outcomes. Our models showed strong discriminative performance and illustrated the predictive value of EUR and endometrial thickness. These findings suggest that ML-based survival analysis models can support more personalized and informative counseling, which may help mitigate treatment discontinuation.

## Supplementary Material

hoag085_Supplementary_Data

## Data Availability

The data underlying this article cannot be shared publicly due to ethical restrictions. The data will be shared on reasonable request to the corresponding author. The code underlying this article is available in the GitLab repository PregnancyOutcome at https://gitlab.kuleuven.be/u0155664/pregnancyoutcome.git.
