# Supplementary material for: Discrete time survival analysis machine learning models for IVF prognosis across multiple cycles
Source: Hum Reprod Open. 2026 Sep 23;2026(4):hoag085. doi: 10.1093/hropen/hoag085 (PMC13693365; doi:10.1093/hropen/hoag085)
Supplement: Supplementary file 1 [file hoag085_supplementary_data.zip › HRO-26-0105_-_Supplementary_File_S1_Final_EO.docx]

**Discrete time survival analysis machine learning models for IVF prognosis across multiple cycles**

TingTing Shao^1,2,*^, Achilleas Ghinis^1,2^, Eline Dancet^3,^, Johanna Devroe^4,5^, Felipe Kenji Nakano^1,2,6^, Celine Vens^1,2^

^1^Department of Public Health and Primary Care, KU Leuven Campus Kulak Kortrijk, Kortrijk, Belgium

^2^Department of Public Health and Primary Care, itec, imec research group at KU Leuven, Kortrijk, Belgium

^3^Department of Public Health and Primary Care, KU Leuven, Leuven, Belgium

^4^Department of Obstetrics and Gynecology, Leuven University Fertility Centre, University Hospital Leuven, Leuven, Belgium

^5^Laboratory of Endometrium, Endometriosis & Reproductive Medicine, Department of Development and Regeneration, KU Leuven, Leuven, Belgium

^6^Computer Science Department, State University of Londrina, Londrina, Brazil

Table of Contents

| Content | Page |
| --- | --- |
| Details about refitted McLernon model | 2-6 |
| Details about BART method | 7 |
| Use case | 7-8 |

# Refitted McLernon Model

## Pretreatment model formula

1. The following Age1 to Age4 equations are first calculated using the female age and then feed into the XB equation below in point 4:

Age1 = age-25

Age2 = (max(age-31,0)^3) – (0.59375 * (max(age-18, 0)^3)) – (0.406250 * (max(age-50, 0)^3 )) + 203.6563

Age3 = (max(age-35, 0)^3 ) – (0.46875 * (max(age-18, 0)^3 )) – (0.53125 * (max(age-50, 0)^3)) + 160.78125

Age4 = (max(age-39,0)^3 ) – (0.34375 * (max(age-18, 0)^3)) – (0.65625 * (max(age-50, 0)^3)) + 117.90625

where ^ means ‘to the power of’

2. Please calculate the following bmi1 to bmi4 equations using the female BMI and then feed into the XB equation below in point 4:

bmi1 = bmi-25

bmi2 = (max(bmi-21.7,0)^3) – (0.83235294 * (max(bmi-16, 0)^3)) – (0.16764706 * (max(bmi-50, 0)^3)) +570.84833

bmi3 =(max(bmi-24.399, 0)^3) – (0.75294119 * (max(bmi-16, 0)^3)) – (0.24705881 * (max(bmi-50, 0)^3)) +548.6781

bmi4 = (max(bmi-28.799,0)^3) – (0.62352943 * (max(bmi-16, 0)^3)) – (0.37647057 * (max(bmi-50, 0)^3)) + 454.55295

where ^ means ‘to the power of’

3. Please calculate the following amh1 to amh4 equations using the AMH measurement and then feed into the XB equation below in point 4:

amh1=amh-2.5

amh2=(max(amh-0.98,0)^3) – (0.93933707 * max(amh-0.01, 0)^3) – (0.06066293 * max(amh-16, 0)^3) + 10.989912

amh3=(max(amh-2.0999, 0)^3) - (0.86929959 * max(amh-0.01, 0)^3) – (0.13070041 * max(amh-16, 0)^3) + 13.356415

amh4=(max(amh-4.03,0)^3) - (0.74859285 * max(amh-0.01, 0)^3) – (0.25140715 * max(amh-16, 0)^3) + 11.556963

where ^ means ‘to the power of’

4. Calculate XB

XB= -0.01585759 + (0.06172204* (Age1)) + (0.00122895* (Age2)) + (-0.00540138* (Age3)) + (0.00587186* (Age4)) + (0.01054925* (bmi1)) + (-0.002679* (bmi2)) + (0.00361132* (bmi3)) + (-0.00121356* (bmi4)) + (0.21484173* (amh1)) + (-0.12160934* (amh2)) + (0.11026825* (amh3)) + (-0.02853336* (amh4)) + (-0.05291497* (OvulDisorder))

XB= -0.00732228+ (0.01113414* (Age1)) + (0.00052396* (Age2)) + (-0.00466203* (Age3)) + (0.00578299* (Age4)) + (0.00601699* (bmi1)) + (-0.00334509* (bmi2)) + (0.0044677* (bmi3)) + (-0.00152398* (bmi4)) + (0.20295926* (amh1)) + (-0.17260625* (amh2)) + (0.15052348* (amh3)) + (-0.03759595* (amh4)) + (-0.09595558* (OvulDisorder)) + (-0.15070413*(maleInfertility))

5. For each couple we want to calculate their probability of live birth after the first, second, and third cycle of IVF:

PCycle1 = exp(XB + 0.36226991)/(1+exp(XB + 0.36226991))

PCycle2 = exp(XB + 0.1189841)/(1+exp(XB + 0.1189841))

PCycle3 = exp(XB - 0.04669854)/(1+exp(XB - 0.04669854))

PCycle4 = exp(XB - 0.33368976)/(1+exp(XB - 0.33368976))

PCycle5 = exp(XB - 0.02940224)/(1+exp(XB - 0.02940224))

PCycle6 = exp(XB - 0.08732107)/(1+exp(XB - 0.08732107))

PCycle1 = exp(XB + 0.29320469)/(1+exp(XB + 0.29320469))

PCycle2 = exp(XB + 0.04403921)/(1+exp(XB + 0.04403921))

PCycle3 = exp(XB -0.05197661)/(1+exp(XB -0.05197661))

PCycle4 = exp(XB -0.21783903)/(1+exp(XB -0.21783903))

PCycle5 = exp(XB -0.02093987)/(1+exp(XB -0.02093987))

PCycle6 = exp(XB -0.05381066)/(1+exp(XB -0.05381066))

6. We then calculate the cumulative probability of a live birth after 1 to 6 cycles:

CumPCycle1 = 1-(1- PCycle1)

CumPCycle2 = 1-((1- PCycle1)*(1- PCycle2))

CumPCycle3 = 1-((1- PCycle1)*(1- PCycle2)*(1- PCycle3))

CumPCycle4 = 1-((1- PCycle1)*(1- PCycle2)*(1- PCycle3))*(1- PCycle4))

CumPCycle5 = 1-((1- PCycle1)*(1- PCycle2)*(1- PCycle3))*(1- PCycle4)) *(1- PCycle5))

CumPCycle6 = 1-((1- PCycle1)*(1- PCycle2)*(1- PCycle3))*(1- PCycle4)) *(1- PCycle5)) *(1- PCycle6))

## Posttreatment model formula

1. The following Age1 to Age4 equations are first calculated using the female age and then feed into the XB equation below in point 4:

Age1 = age-25

Age2 = (max(age-33,0)^3) – (0.5666666 * (max(age-20, 0)^3)) – (0.43333334 * (max(age-50, 0)^3 )) + 70.833336

Age3 = (max(age-37, 0)^3 ) – (0.43333334 * (max(age-20, 0)^3 )) – (0.566666 * (max(age-50, 0)^3)) + 54.166668

Age4 = (max(age-40,0)^3 ) – (0.33333334 * (max(age-20, 0)^3)) – (0.6666666 * (max(age-50, 0)^3)) + 41.666668

where ^ means ‘to the power of’

2. Please calculate the following bmi1 to bmi4 equations using the female BMI and then feed into the XB equation below in point 4:

bmi1 = bmi-25

bmi2 = (max(bmi-21.7,0)^3) – (0.83679527 * (max(bmi-16.2, 0)^3)) – (0.16320473 * (max(bmi-49.9, 0)^3)) +534.31543

bmi3 = (max(bmi-24.5, 0)^3) – (0.75370926 * (max(bmi-16.2, 0)^3)) – (0.24629074 * (max(bmi-49.9, 0)^3)) +513.50659

bmi4 = (max(bmi-29,0)^3) – (0.62017804 * (max(bmi-16.2, 0)^3)) – (0.37982196 * (max(bmi-49.9, 0)^3)) + 422.63388

where ^ means ‘to the power of’

3. Please calculate the following Retr1 to Retr4 equations using the number of Eggs collected and then feed into the XB equation below in point 4:

gen Retr1=Retr-9

gen Retr2=(max(Retr-2,0)^3) – (0.93103451 * (max(Retr-0, 0)^3)) – (0.06896549 * (max(Retr-29, 0)^3)) + 335.72415

gen Retr3=(max(Retr-7, 0)^3) - (0.75862068 * (max(Retr-0, 0)^3)) – (0.24137932 * (max(Retr-29, 0)^3)) + 545.03448

gen Retr4=(max(Retr-13,0)^3) - (0.58620691 * (max(Retr-0, 0)^3)) – (0.41379309 * (max(Retr-29, 0)^3)) + 427.34482

where ^ means ‘to the power of’

4. Calculate XB

XB = -0.14822068

+ (-0.041263 * (Age1)) + (-0.00347325 * (Age2)) + (0.00896319 * (Age3)) + (-0.00584328 * (Age4))

+ (-0.00547436 * (bmi1)) + (-0.00285918 * (bmi2)) + (0.003437 * (bmi3)) + (-0.00101838* (bmi4))

+ (0.14227969 * (Retr1)) + (-0.0008879 * (Retr2)) + (0.00093414 * (Retr3)) + (-0.00019994 * (Retr4)) + (-0.0772825 * (OvulDisorder))

XB = -0.17819359

+ (2.29853040e-02* (Age1)) + (-1.22027550e-03* (Age2)) + (2.24173728e-03* (Age3)) + (-3.90831931e-04* (Age4))

+ (8.23155054e-03* (bmi1)) + (-3.21847458e-03* (bmi2)) + (4.16122021e-03* (bmi3)) + (-1.39665181e-03* (bmi4))

+ (1.42859130e-01* (Retr1)) + (-7.23764639e-04* (Retr2)) + (8.07604453e-04* (Retr3)) + (-1.55003642e-04* (Retr4)) + (-7.20154733e-02* (OvulDisorder) + (-2.22612977e-01*(maleInfertility)))

5. For each couple we want to calculate their probability of live birth after the first, second, and third cycle of IVF:

PCycle1 = exp(XB+0.16578684)/(1+exp(XB+0.16578684))

PCycle2 = exp(XB -0.02809679)/(1+exp(XB -0.02809679))

PCycle3 = exp(XB-0.0553473)/(1+exp(XB-0.0553473))

PCycle4 = exp(XB- 0.1881462)/(1+exp(XB- 0.1881462))

PCycle5 = exp(XB - 0.00921559)/(1+exp(XB - 0.00921559))

PCycle6 = exp(XB- 0.03320163)/(1+exp(XB- 0.03320163))

PCycle1 = exp(XB+ 1.66855800e-01)/(1+exp(XB+ 1.66855800e-01))

PCycle2 = exp(XB -1.22615292e-02)/(1+exp(XB -1.22615292e-02))

PCycle3 = exp(XB -4.69202217e-02)/(1+exp(XB -4.69202217e-02))

PCycle4 = exp(XB -1.84754091e-01)/(1+exp(XB -1.84754091e-01))

PCycle5 = exp(XB -7.95956773e-03)/(1+exp(XB -7.95956773e-03))

PCycle6 = exp(XB -3.33247433e-02)/(1+exp(XB -3.33247433e-02))

6. We then calculate the cumulative probability of a live birth after 2, and 3 cycles:

CumPCycle1 = 1-(1- PCycle1)

CumPCycle2 = 1-((1- PCycle1)*(1- PCycle2))

CumPCycle3 = 1-((1- PCycle1)*(1- PCycle2)*(1- PCycle3))

CumPCycle4 = 1-((1- PCycle1)*(1- PCycle2)*(1- PCycle3))*(1- PCycle4))

CumPCycle5 = 1-((1- PCycle1)*(1- PCycle2)*(1- PCycle3))*(1- PCycle4)) *(1- PCycle5))

CumPCycle6 = 1-((1- PCycle1)*(1- PCycle2)*(1- PCycle3))*(1- PCycle4)) *(1- PCycle5)) *(1- PCycle6))

# Bayesian Additive Regression Trees

As described by (Chipman *et al.*, 2010), A single tree in BART was expressed as$T_{i}=g\left( x; T_{i}, M_{i} \right) + \epsilon, \epsilon\sim N\left( 0, \sigma^{2} \right)$, with M_i_ denoted as a set of functional values associated with the b terminal nodes $M_{i}=\left\{ \mu_{1}, \mu_{2}, \ldots, \mu_{b} \right\}$, and $\epsilon$being a Gaussian error with mean 0 and variance $\sigma$. Therefore, BART ensemble tree model with m trees in total is determined by $\left( T_{1}, M_{1} \right), \left( T_{2}, M_{2} \right), \ldots, \left( T_{m}, M_{m} \right)$ and $\sigma$, and there are two priors to decide the tree complexity: prior on tree structure ($T_{j}$), and prior on node parameter in each tree ($\mu_{ij}$|$T_{j}$).

$T_{j}$ prior considers three aspects: (1) the probability of a node at depth d is non-terminal is calculated as $\alpha\left( 1+d \right)^{-\zeta}$ with $\alpha$and $\zeta$ denoted as base and power parameter, respectively; (2) The probability of selecting a feature to split is uniformly distributed; (3) The probability of selecting a cutoff given a feature is uniformly distributed. On the other hand, prior for the functional value in each terminal node in a tree ($\mu_{ij}\sim N\left( 0, \sigma_{\mu}^{2} \right), \sigma_{\mu} = \frac{0.5}{k\sqrt{m}}$), has an effect of shrinking the tree parameters $\mu_{ij}$towards zero, limiting the contribution of the individual tree. During Bayesian backfitting, the posterior probability of a updated tree is compared with the previous tree, and decides whether to accept a new tree structure or not.

# Use case

As a demonstration of the information offered by the BART model, we used an example from the validation dataset. Supplementary Table S6 summarizes the characteristics of the couple, who achieved success after their first IVF cycle. The female partner was 34.1 years old with an imputed AMH level of 2.83 ng/mL. Post-treatment measurements revealed favorable prognostic indicators: EM of 11.2 mm and 12 oocytes retrieved.

Supplementary Figure S9 displays the predicted survival curves for the couple derived from both pre-treatment and post-treatment models. Before treatment initiation, the model estimated a patient-specific success rate of 35.5% for the first cycle, slightly exceeding the population average of 33.9% (95% CI: 31.5%–36.4%). The pre-treatment model predicted cumulative success rates of 35.5% (95% CI: 28.1%–43.5%) over cycle 1, 55.8% (46.2%–65.9%) over cycle 2, and 68.5% (58.6%–78.4%) over cycle 3.

After incorporating information obtained from the first cycle treatment, the post-treatment model substantially revised the prognosis of the first cycle to 61.1%. The conditional success rate at each cycle presuming no success occurred before showed a gradual decline except at the 5^th^ cycle: 61.1% (cycle 1), 58.5% (cycle 2), 56.4% (cycle 3), 49.3% (cycle 4), 51.8% (cycle 5), and 49.4% (cycle 6). As expected, cumulative success rates increased progressively: 61.1% (95% CI: 48.9%–73.4%), 83.5% (73.0%–92.0%), 92.5% (85.2%–97.6%), 96.0% (90.6%–99.1%), 97.9% (94.2%–99.7%), and 98.8% (96.4%–99.9%) over one to six cycles. For individual estimates, the quantification of the prediction uncertainties is provided, which enhances the model reliability and allows room for clinicians to interpret the estimates in the context of their own experience and in-person contact with the patients.

# Reference

Chipman HA, George EI, McCulloch RE. BART: Bayesian additive regression trees. *Ann Appl Stat* [Internet] 2010;**4**: 266-298.
